# Enriching rice with Zn and Fe while minimizing Cd risk

**DOI:** 10.3389/fpls.2015.00121

**Published:** 2015-03-12

**Authors:** Inez H. Slamet-Loedin, Sarah E. Johnson-Beebout, Somayanda Impa, Nikolaos Tsakirpaloglou

**Affiliations:** ^1^Plant Breeding, Genetics, and Biotechnology Division, International Rice Research InstituteManila, Philippines; ^2^Crop and Environmental Sciences Division, International Rice Research InstituteManila, Philippines

**Keywords:** rice, Cd contamination, genetic biofortification, risk mitigation, Zn enriched rice, Fe enriched rice, agronomic biofortification

## Abstract

Enriching iron (Fe) and zinc (Zn) content in rice grains, while minimizing cadmium (Cd) levels, is important for human health and nutrition. Natural genetic variation in rice grain Zn enables Zn-biofortification through conventional breeding, but limited natural Fe variation has led to a need for genetic modification approaches, including over-expressing genes responsible for Fe storage, chelators, and transporters. Generally, Cd uptake and allocation is associated with divalent metal cations (including Fe and Zn) transporters, but the details of this process are still unknown in rice. In addition to genetic variation, metal uptake is sometimes limited by its bioavailability in the soil. The availability of Fe, Zn, and Cd for plant uptake varies widely depending on soil redox potential. The typical practice of flooding rice increases Fe while decreasing Zn and Cd availability. On the other hand, moderate soil drying improves Zn uptake but also increases Cd and decreases Fe uptake. Use of Zn- or Fe-containing fertilizers complements breeding efforts by providing sufficient metals for plant uptake. In addition, the timing of nitrogen fertilization has also been shown to affect metal accumulation in grains. The purpose of this mini-review is to identify knowledge gaps and prioritize strategies for improving the nutritional value and safety of rice.

## INTRODUCTION

Iron (Fe) and zinc (Zn) deficiencies affect more than two billion people globally (McLean et al., 2009; Wessells and Brown, 2012). Fe-deficiency anemia can cause impaired cognitive and physical development in children and reduction of daily productivity in adults (Black et al., 2013; Stevens et al., 2013). Recently, low maternal Fe intake has been linked to autism spectrum disorder in their offspring (Schmidt et al., 2014). Adequate Zn nutrition is also important for child growth, immune function, and neurobehavioral development (Wessells and Brown, 2012). Biofortification, defined as increasing the micronutrient content in staple food (Bouis et al., 2011), has the potential to combat Fe and Zn deficiencies, but it is important to ensure low presence of undesirable toxic metals. Because cadmium (Cd) tends to accumulate in kidneys throughout a person’s life, there is concern that regular consumption of rice with even moderate Cd concentration may result in health problems, especially for people who consume rice as a staple food (Meharg et al., 2013). Here we review the genetics and nutrient management approaches to increasing Fe and Zn and minimizing possible Cd contamination.

## CONVENTIONAL, MARKER ASSISTED AND TRANSGENIC BREEDING APPROACHES FOR BIOFORTIFICATION TO ENHANCE Fe AND Zn CONCENTRATIONS IN RICE

Nutritional studies suggested that 24–28 mg kg^-1^ Zn and 13 mg kg^-1^ Fe concentration in polished grain is essential to reach the 30% of human estimated average requirement (Bouis et al., 2011). Based on this, rice germplasm diversity has been exploited to breed Zn-dense varieties conventionally (Graham et al., 1999). Two Zn-enriched varieties, reaching up to 19 and 24 mg kg^-1^ Zn in rice grains, have been released by Bangladesh Rice Research Institute (BRRI) in collaboration with the International Rice Research Institute (IRRI) under the HarvestPlus project. Identification of quantitative trait loci (QTLs) for low to moderate Zn enhancement in the existing rice germplasm were reported (Stangoulis et al., 2006; Anuradha et al., 2012; Neelamraju et al., 2012). In addition, genome wide association mapping revealed several loci associated with Zn levels in grains (Norton et al., 2014). However, large effect Zn QTLs (≥30% phenotypic variation) have not been identified yet. Conventional breeding efforts for developing Fe-enriched polished rice have not progressed effectively due to limited variation of Fe concentration in polished rice. Evaluation of more than 20,000 rice accessions from Asia, Latin America, and the Caribbean for Fe and Zn concentration revealed a maximum of only 8 mg kg^-1^ in polished grains (Gregorio et al., 2000; Graham, 2003; Martínez et al., 2010). Most Fe and Zn are concentrated in the aleurone layers of rice bran. There are between 1 and 5 aleurone layers in different rice accessions (del Rosario et al., 1968); therefore, the high Fe levels in unpolished grains can be due to thickness of the bran layers. Conventional breeding has so far been unsuccessful in the development of Fe-enriched polished rice (Bashir et al., 2013a).

Transgenic approaches to enhance Fe in the starchy endosperm were first explored more than a decade ago (Goto et al., 1999). Since then, researchers have attempted to increase Fe content in rice endosperm by overexpressing genes involved in Fe uptake from the soil and translocation from roots, shoot, flag leaf to grains, and by increasing the efficiency of Fe storage proteins (**Table 1**; Kobayashi and Nishizawa, 2012; Lee et al., 2012; Bashir et al., 2013a; Masuda et al., 2013a). Among these studies, the concomitant increase in Fe and Zn content in rice grains was obtained by the overexpression or activation of the *NAS* (nicotianamine synthase) genes, either *in solo* or in combination with other transporters or Fe storage genes (**Table 1**). *NAS* catalyzes the synthesis of the divalent metal chelator nicotianamine acid (NA) from the precursor molecule 2’-deoxymugeneic acid (MA). Constitutive expression of *OsNAS2* resulted in increased Fe concentration as high as 19 mg kg^-1^and Zn concentration to as high as 76 mg kg^-1^ within the endosperm of polished rice grains (Johnson et al., 2011). On the other hand, the baseline of *O. japonica* cv. Nipponbare in this study is 4 mg kg^-1^ Fe, which is higher than other studies employing *japonica* accessions (**Table 1**), possibly due to a favorable micro-environment. Combinations of genes involved in chelating, transporting or storing Fe significantly enhanced Fe concentration to reach polished grain concentration as high as 8–9 mg kg^-1^ (Masuda et al., 2012, 2013b; Aung et al., 2013). These studies also demonstrated the stability of the trait over multiple plant generations; nevertheless, reaching the recommended target level still remains a challenge. Furthermore, to accelerate the farmers’ adoption and consumers’ acceptance, Oliva et al. (2014) generated phytoferritin over-expressor events in popular *indica* variety without selectable marker genes; however, the level of Fe was not sufficient to reach the target.

**Table 1:** Summary of transgenic approaches to improve Iron (Fe)/Zinc (Zn) concentrations in rice grains and to reduce Cadmium (Cd).

Gene	Promoter	Cultivar	Growth conditions	Generation of seeds	Fe concentration (ppm)	Fold increase in Fe	Zn concentration (ppm)	Fold increase in Zn	Effect on Cd concentration in the grains	Reference
**(A) Overexpression approaches**
**(1) Brown seeds**
*SoyferH1*	*OsGluBI*	*Japonica* cv. Kitaake	Greenhouse	T1	∼38.0	3.0	n.a.	n.a.	n.a.	Goto et al. (1999)
*SoyFerH1*	*OsGlu ; OsGtbl*	*Japonica* cv. Kiktake	Sreenhouse	T3 to T6	up to 27.0	3.0	up to 46.0	1.1	Similar to WT	Qu et al. (2005)
*PyFerritin*+rgMT	*OsGluBl*	*Japonica cv.* Taipei 309	Greenhouse	T1	∼22.0	2.0	n.a.	n.a.	n.a.	Lucca et al. (2002)
*TOM1*	*CaMV 35S*	*Japonica* cv. Tsukinohikari	Hydroponic	T1	∼18.0	1.2	∼45.0	1.6	n.a.	Nozoye et al. (2011)
*SoyferH1*	*ZmUbil*	*Indica* cv. M12	Greenhouse	T2 homozygous	∼18.0	No significant increase	n.a.	n.a.	n.a.	Drakakaki et al. (2000)
*OsIRO2*	*CaMV 35S*	*Japonica* cv. Tsukinohikari	Greenhouse	T1	up to 15.5	2.8	up to 13.0	1.4		Ogo et al. (2011)
			(Calcareous soil)							
*OsYSL15*	*OsAcinl*	*Japonica* cv. Dongjin	Paddy field	T1	∼14.0	1.1	∼23.5	1.0	n.a.	Lee et al. (2009a)
*OsIRT1*	*ZmUbil*	*Japonica* cv. Dongjin	Paddy field	T3 homozygous	∼12.0	1.1	∼22	1.1	Similar to WT (roots and shoots)	Lee and An (2009)
*HvNAS1, HvNAS1+HvNAAT, IDS3*	Genomic fragments	*Japonica* cv. Tsukinohikari	Paddy field (Calcareous soil)	T1	up to 7.3	1.2	up to 15.3	1.4	n.a.	Suzuki et al. (2008)
*OsNAS1*	*OsGluBl*	*Japonica* cv. Xiushui 110	field	??	∼5.0	1.0	∼30.0	1.3	n.a.	Zheng et al. (2010)
**(2) Milled seeds**
*SoyFerH1*	*OsGluBl*	*Indica* cv. IR68144	Screenhouse	T2		3.7	∼55.0	1.4	n.a.	Vasconcelos et al. (2003)
*SoyFerH1*		*Indica* cv. Swama	Greenhouse	BC2F5	up to 16.0	2.5	up to 27.5	1.5	n.a.	Paul et al. (2014)
*OsFer2*	*OsGluA2*	Basmati rice (Indica cv. Pusa-Sugandh II)	Greenhouse	T3	up to 15.9	2.1	up to 30.75	1.4	n.a.	Paul et al. (2012)
*OsNAS3*	Activation tagging	*Japonica* cv. Dongjin	Greenhouse	T1	∼12.0	2.6	∼35.0	2.2	Similar to *WT*	Lee et al. (2009b)
*OsNAS2*	Activation tagging	*Japonica* cv. Dongjin	Greenhouse	??	∼10.0	3.0	∼42.0	2.7	Similar to WT	Lee et al. (2011, 2012)
**(3) Polished seeds**
*OsNAS1, OsNAS2, OsNAS3*	*CaMV 35S*	*Japonica* cv. Nipponbare	Glasshouse	T1	up to 19.0	2.2, 4.2, 2.2	up to 76.0	1.4, 2.2, 1,4	n.a.	Johnson et al. (2011)
*SoyFerH1*	*GluB1*	*Indica* cv. BR29	Greenhouse	T3	up to 9.2	2.4	n.a.	n.a.	n.a.	Khalekuzzaman et al. (2006)
*HvNAS1*	*CaMV 35S*	*Japonica* cv. Tsukinohikari	Greenhouse	T2	∼8.5	2.5	∼28.0	1.5	n.a.	Higuchi et al. (2001), Masuda et al. (2009)
*SoyFerH1, SoyFerH2, OsFer1C, OsFer2C*	*CluBI and GluB4, CluB1 and GluB4, CluB1 and GluB4, CluB1 and GluB4*	*Indica* cv. 1R64	Greenhouse	T4	up to 7.6	2.3	n.a.	n.a.	n.a.	Oliva et al. (2014)
*SoyFerH1, SoyFerH2*	*CluB1 and GluB4, CluB1*	*Indica* cv. IR64	Greenhouse	T5	up to 5.9	1.8	n.a.	n.a.	n.a.	
*HvNAS1*	*OsActinl*	*Japonica* cv. Tsukinohikari	Greenhouse	T1	∼7.5	3.4	∼35.0	2.3	n.a.	Masuda et al. (2009)
*OsYSL2*	*OsSUT1*	*Japonica* cv. Tsukinohikari	Glasshouse	T1	∼7.5	4.4	n.a.	n.a.	n.a.	Ishimaru et al. (2010)
*AtNAS1+, Pvferritin*+*, Afphytase*	*CaMV 35S, Glbl, Glbl*	*Japonica* cv. Taipei 309	Hydroponic	T1	∼7.0	6.3	∼33.0	1.6	n.a.	Wirth et al. (2009)
*OsYSL2+, SoyFerH2+, HvNAS1*	*OsSUT1 and Glbl, GluB1 and Glbl, OsActl*	*Japonica* cv. Tsukinohikari	Greenhouse (and paddy field)	T2 (and 73)	up to 7.0	6 (and 4)	∼20.0	1.6	Similar to WT	Masuda et al. (2012)
*SoyFerH2+, HvNAS1+, OsYSL2*	*OsGluBl and OsG1b, OsActinl, OsSUT1 and OsGtbl*	*Tropical Japonica* cv. Paw San Yin (Myanmar high quality rice)	Greenhouse	T1 (and T2)	6.3 (up to 5.02)	2 (up to 3.4)	34.2 (up to 39.2)	1.1 (up to 1.3)	.1,	Aung et al. (2013)
*SoyFerH2, HvNAS1, HvNAAT-A, -B and IDS3 genome fragments*	*OsGluBl, OsGtbl*	*Japonica* cv. Tsukinohikari	Greenhouse Greenhouse (calcareous soil)	T3 T3	up to 4.0 up to 5.0	2.6 2.5	up to 31 up to 25.0	1.5 1.4	n.a. n.a.	Masuda et al. (2013b)
*HyNAS1, HyNAS1+HyNAAT, IDS3*	Genomic fragments	*Japonica* cv. Tsukinohikari	Paddy field (Andosolsoi)	T1	1.11, 1.19, 1.49	1.0, 1.1, 1.4	11.3, 11.9, 14.3	1.0, 1.1, 1.3	n.a.	Masuda et al. (2008)
**(B) Silencing approaches**
*OsVIT*	T-DNA mutant	*Japonica* cv. Zhonghual 1	Paddy field		∼16	∼1.4	∼31	∼1.2	↑	Zhang et al. (2012)
			(0.55 ppm Cd)							
*OsVIT2*	T-DNA mutant	*Japonica* cv. Dongjin	Paddy field (0.55 ppm Cd)		∼14	∼1.5	∼30	∼1.3	↑	Zhang et al. (2012)
*OsNRAMP5*	RNAi	*Japonica* cv. Tsukinohikari	glasshouse (10μM Cd)		n.a.	n.a.	n.a.	n.a.	↓	Ishimaru et al. (2012)^∗^

*Silencing of *OsNRAMP5* (Natural Resistance-Associated Macrophage Protein 5) has also been obtained through ion-beam irradiation (Ishikawa et al., 2012). Different approaches have been grouped based the transgenic over expression vs. down regulation (silencing) approaches, and available Fe/Zn data (polished grain or brown rice). The arrows (↑) or (↓) indicate the increase/decrease of Cd concentration in rice grains.

The average of 2 mg kg^-1^ Fe in well-polished rice g rains is the general baseline in popular varieties (Bouis et al., 2011). However, there was a marked variation in the baseline of Fe concentration between genotypes used in the studies described in **Table 1**. Such variation could be due to differences in the milling degree of rice grains, the respective genotypes as such, or the growth conditions, and fertilizer applications. In addition, Fe measurement is also highly prone to contamination during seed processing, milling, and analytical process.

Most Fe biofortification studies were conducted under favorable glasshouse conditions, with only limited studies performed under field conditions (Masuda et al., 2008, 2012). In the first study, moderate increases of 1.40-fold for Fe and 1.35-fold for Zn concentrations of transgenic polished rice grains were observed compared to the control (Masuda et al., 2008). In the second study, a significant decrease (up to 50%) was observed in the Fe concentration in polished grains in the subsequent generation of T_3_ homozygous plants grown under paddy field conditions (4 mg kg^-1^) compared to the earlier generation grown under the glasshouse condition (Masuda et al., 2012) that reached up to 7–8 mg kg^-1^ (six times the concentration of the wild type control).

Among genetic improvement options for increasing rice grain Fe and Zn, we recommend the prioritization of the sink and source strategy (Wirth et al., 2009; Masuda et al., 2013a). However, despite the fast progress, reaching the nutritionist recommended target level of 13 mg kg^-1^ for Fe under field conditions (Bouis et al., 2011) still remains a challenge (Bashir et al., 2013a). Therefore, to enhance Fe and Zn content in polished rice grains, the expression of most optimum orthologoues of chelator(s), transporter genes and iron storage genes still needs to be evaluated. In addition, for product development, data on the transgene copy number is required.

## GENETICS OF CADMIUM UPTAKE

In general, *indica* varieties accumulated higher Cd concentrations compared to *japonica* in Cd-polluted soils or in hydroponic solution with high Cd (Arao and Ishikawa, 2006). The physiological mechanisms for Cd uptake and its translocation to shoots in rice have been associated with several chemically related metal ions (Kim et al., 2002; Arao and Ishikawa, 2006; Uraguchi and Fujiwara, 2012). Absorption of Cd in hydroponically grown Fe-deficient plants was thought to be mediated through the Fe-uptake system, particularly through the *OsIRT1* and *OsIRT2* genes (Nakanishi et al., 2006). *OsNRAMP1* (Natural Resistance-Associated Macrophage Protein 1) is another transporter protein shown to be related to the absorption of Cd in rice roots (Takahashi et al., 2011). Functional analysis of the gene confirmed its expression in roots, whilst the protein was localized in the plasma membrane, indicating its role in Cd absorbance and transport (Takahashi et al., 2011).

Recently, it has been demonstrated that the *OsNRAMP5* gene in rice acts as a major transporter of Cd and Mn in the roots (Ishikawa et al., 2012; Sasaki et al., 2012). Expression analysis showed that its presence was restricted to roots, as well as in tissues around the xylem (Ishimaru et al., 2012; Sasaki et al., 2012). In addition, extensive analysis of silencing, insertion knock-out plants, and ion-beam irradiation mutants confirmed the role of *OsNRAMP5* in reducing the Cd accumulation both in straw and in grains to negligible levels, even when grown in Cd-contaminated paddy fields (Ishikawa et al., 2012; Ishimaru et al., 2012; Sasaki et al., 2012). Using a different approach, hydroponic and soil culture experiments suggested root-to-shoot Cd translocation via the xylem as the major physiological process for determining grain Cd accumulation in rice (Uraguchi et al., 2009). Analysis of mapping populations for identification of QTLs related to Cd accumulation in rice grains indicated the presence of a genetic locus in chromosome 7 (*qGCd7*; Ishikawa et al., 2005, 2010). This QTL was shown to be specific to Cd since it was not related to the absorption/translocation of other metal cations or to any agronomic characteristics. Fine mapping of the *qGCd7* resulted in the identification of *OsHMA3*, a gene responsible for limiting the root-to-shoot translocation of Cd by selectively sequestering it within the vacuoles (Ueno et al., 2010; Miyadate et al., 2011). *OsHMA2*, a close homolog of *OsHMA3*, has also been shown to be involved in the root-to-shoot translocation of Cd in rice plants, through the xylem network (Satoh-Nagasawa et al., 2012; Takahashi et al., 2012).

Furthermore, Uraguchi et al. (2011) proposed a different route for reducing Cd within the rice grains. The identification of the low-affinity cation transporter (*OsLCT1*) reduced the Cd accumulation within rice grains by significantly decreasing its phloem-mediated transport. Suppression of *OsLCT1* did not have any negative effect on the content of other metal ions in the grains, indicating its specificity for Cd (Uraguchi et al., 2011, 2014). Among genetic strategies for decreasing Cd concentration in rice, we recommend prioritization of strategies reducing the sequestration of Cd in roots, such as down-regulation of *OsNRAMP5*. This has been achieved recently by RNAi transgenic approach and mutation technologies (Ishikawa et al., 2012; Ishimaru et al., 2012).

## HAS CADMIUM BEEN ACCUMULATED IN ENRICHED Fe/Zn RICE?

Conventional breeding lines with enriched grain Zn have not been reported to contain elevated Cd. The fact that Fe/Zn-biofortification by transgenic approaches exploited different transporter genes (**Table 1**) raises the possibility of Cd accumulation because Zn-associated transporters often co-transport Zn-mimic Cd (Olsen and Palmgren, 2014). The upper limit of Cd set by FAO/WHO in rice grain is 0.4 mg kg^-1^ (Codex Alimentarius, 2010). The transgenic approaches that tended to simultaneously increase grain Zn as well as Fe were the ones involving the *NAS* family genes (**Table 1**). However, assessment of seedlings of *OsNAS3* activation tag lines and its wild counterpart in plant growth medium with elevated Cd showed no difference in Cd level amongst different germplasm and tissues (Lee and An, 2009; Lee et al., 2009b, 2011), suggesting the specificity of NA to Zn over Cd (Olsen and Palmgren, 2014). In addition, a 20% reduction in the Cd accumulation was identified in T_2_ polished grains compared to the non-transgenic counterparts expressing transporters and phytoferritin genes (Aung et al., 2013). Another transporter protein, *OsIRT1*, has been suggested to be involved in the Fe and Cd uptake pathway earlier (Nakanishi et al., 2006). However, the translocation of excess Cd from the roots to shoots was minimal. Recent studies in *osvit1* and *osvit2* T-DNA knock out mutants reported some increase in Cd level in rice grains (Zhang et al., 2012). To date only one report on transgenic biofortified rice shows a slight increase in the Cd levels (Zhang et al., 2012), whilst there have been no reports yet on the grain Cd level on the Zn-enriched conventional breeding lines. In all the reported approaches, the acquired Cd concentrations were significantly lower than the threshold toxic levels for the polished rice grains.

## MANAGEMENT AND ENVIRONMENT EFFECTS ON Fe, Zn, and Cd UPTAKE IN RICE

The performance of biofortified genotypes is often restricted due to low available pools of Zn or Fe in soil. Under these conditions, enriching Fe or Zn concentration in grains through either fertilization or water management, called agronomic biofortification, is a short term strategy which would complement the breeding programs. Some of these management and environment effects have also been shown to change Cd uptake patterns.

## WATER MANAGEMENT

Irrigation management in rice strongly influences soil redox potential, which affects the availability of Fe, Zn, and Cd. Rice was domesticated under flooded conditions, and it is still grown with continuous soil submergence in many places. However, for a variety of reasons, rice is now produced across the entire range of irrigation management options, including fields which are always aerobic, always anaerobic, and many variations along the aerobic-anaerobic spectrum (Bouman et al., 2007). Because socioeconomic drivers are so important in designing irrigation systems, it seems unlikely that farmers would choose irrigation options solely for the purpose of changing the soil availability of Fe, Zn, or Cd. Therefore, we need to understand the effect that water management has on the benefits and risks of enriching grains with metals, even though the opportunities for managing the risks this way are limited.

**FIGURE 1:**
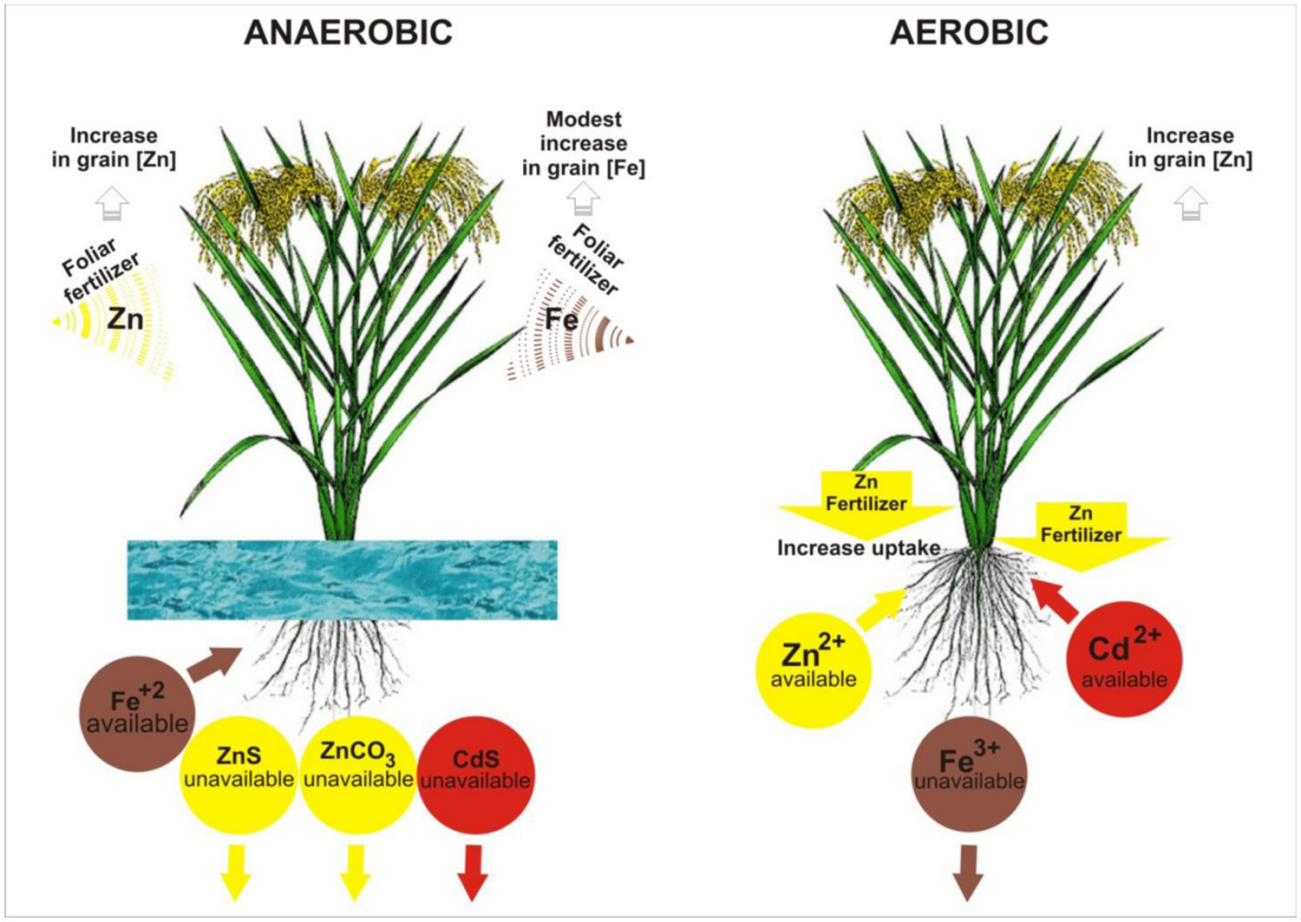
**Illustration of water and fertilizer managements and their effects on zinc (Zn), iron (Fe), and cadmium (Cd) uptake and accumulation in rice grain.** The arrows (↓) under the compounds indicate precipitation.

As a soil changes from aerobic to anaerobic conditions after flooding, Fe- oxides are dissolved when the Fe^3+^ is reduced to Fe^2+^ (**Figure 1**), which weakens the oxide stability and increases its water-solubility (Kirk, 2004). This releases much more Fe into the soil solution, so flooded soil nearly always has sufficient Fe for plant uptake, and rice has therefore become somewhat adapted to Fe toxicity. Most rice plants have mechanisms to prevent excessive uptake of Fe. Anti-oxidative mechanisms, including induction of ferritin gene, have been reported as one of the plant mechanisms against excessive plant endogenous Fe^2+^ (Briat et al., 2010). In contrast, in aerobic soils, Fe deficiency can occur (Zuo and Zhang, 2011), while Zn and Cd both tend to be more available in this soil. Both elements are predominantly present in the +2 oxidation state, regardless of soil redox potential, so the effect of flooding is indirect (rather than direct as with Fe). The availability of Zn decreases with flooding due to precipitation (**Figure 1**) as insoluble zinc sulphide (after sulfate is reduced to sulphide, Bostick et al., 2001) or as insoluble carbonate mixtures (after decomposing organic matter causes an increase in the partial pressure of carbon dioxide in soil solution, Kirk, 2004). Cadmium behaves similarly to Zn (Du Laing et al., 2009). In summary, changing a soil from aerobic to anaerobic conditions by flooding will increase Fe availability and suppress Cd, but will also decrease Zn availability (**Figure 1**). The possibility of managing irrigation to optimize the plant uptake of Fe, Zn, and Cd simultaneously is negligible.

## FERTILIZATION OPTIONS

Most evidence has shown that applying Fe or Zn fertilizers to the soil is ineffective at increasing grain Fe or Zn in rice. Under aerobic water management, the soil-applied Fe (usually in the form of Fe^2+^, either chelated or as a sulfate salt) is rapidly converted to unavailable Fe^3+^, and hence, foliar application is a better option to overcome Fe deficiency and to increase grain Fe and its bioavailability in rice (Wei et al., 2012a). Under anaerobic water management, Fe^2+^ is readily available to rice plants (**Figure 1**), so no fertilization is needed. Application of Zn at 5–25 kg Zn ha^-1^ as zinc sulfate incorporated to the soil before flooding or after transplanting is the most common Zn fertilizer recommendation for rice (Dobermann and Fairhurst, 2000). However, soil-applied zinc sulfate has often been unsuccessful in improving grain Zn concentration and yield under flooded paddy due to redox induced fixation of applied Zn (Srivastava et al., 1999; Johnson-Beebout et al., 2009). In rice, positive effects of soil Zn fertilization on grain Zn have been noticed primarily with aerobic water management (Wang et al., 2014). On the other hand, foliar Zn application has been more effective in improving grain Zn concentration in flooded rice compared to soil Zn fertilization (Wissuwa et al., 2008; Wirth et al., 2009). Zn and Fe fertilization strategies and its effects on the uptake and accumulation of Zn, Fe, and Cd in rice are illustrated in **Figure 1**.

Although foliar application of Fe or Zn is more promising than soil application for enhancing grain Fe or Zn, the efficiency of foliar applied Fe or Zn varies depending on the time of fertilization, source of Zn fertilization and ability of genotypes to remobilize Zn or Fe from source tissues to grain (Karak et al., 2006; Cakmak, 2009; Wei et al., 2012b). Late season foliar application of Zn or Fe at flowering or at early grain filling stage is more effective in improving grain Zn or Fe, respectively, than early season application (Phattarakul et al., 2012; Mabesa et al., 2013). Though the levels of Zn and Fe in grains are positively related, fertilization of one element did not affect the grain concentration of the other (Cakmak et al., 2010; Wei et al., 2012a,b). However, foliar fertilization of combined Fe and Zn fertilizers enhanced both grain-Fe and -Zn content without any antagonistic effects (Wei et al., 2012a). Among fertilization strategies for flooded rice, the most likely to succeed is a combined foliar Zn and Fe spray soon after flowering or at early grain filling stage, and it is important to study how to make foliar fertilizers more effective.

Optimized management of N fertilizer could improve grain Fe and Zn, as indicated by a strong correlation of seed Fe and Zn with *N* in several crop species under sufficient Zn supply (Zhang et al., 2008; Cakmak et al., 2010; Kutman et al., 2010) Better N nutrition promotes protein synthesis, which is a major sink for Fe and Zn, and enhances the expression Zn and Fe transporter proteins, such as ZIP family transporters (Cakmak et al., 2010). Better N nutrition may also enhance the production of other nitrogenous compounds such as NA and deoxymugineic acid (DMA), and YSL proteins involved in Zn transport within the plant (Haydon and Cobbett, 2007; Curie et al., 2009). Under high N supply, vegetative growth is enhanced and plants remain green for a longer time, resulting in longer grain filling periods, and delayed senescence (Kutman et al., 2010). However, under low Zn conditions, increased biomass production induced by optimal N fertilization can decrease grain Zn concentration due to biological dilution (Zhang et al., 2008; Kutman et al., 2012). In summary, it is always important to optimize N fertilization in rice production, but there is not very much scope for adjusting N management for the purpose of biofortification.

Phosphate fertilizers are major sources of Cd input in agricultural land and in cereal crops (Eriksson, 1990; He and Singh, 1993; Gao et al., 2010). They can contain significant amounts of Cd due to its presence in the rock phosphate used for production (Williams and David, 1973). However, once recognized, these relatively high-Cd phosphate rock sources have been avoided in the production of fertilizer, so there is very little evidence of actual P-fertilizer-related Cd uptake in rice. The effect of Zn fertilization on Cd uptake by plants is highly dependent on the soil Cd and Zn concentrations. Higher biomass accumulation under high NPK fertilization, results in enhanced Cd uptake but may either increase or decrease concentration, depending on the balance of fertilizer effects on crop growth, root distribution, and Cd availability. This could be a useful strategy for phytoremediation but not for cereal production. Increase in Cd uptake under higher rate of fertilization than lower rate of fertilization (Singh, 1990), suggests that efficient management of fertilizers is necessary to keep a control on Cd accumulation in agricultural crops.

## IMPROVING IRON AND ZINC NUTRITION, AND MITIGATING CADMIUM TOXICITY RISK THROUGH GENETICS AND MANAGEMENT APPROACHES

Biofortified rice has a potential to reach areas that currently could not be reached by other interventions since rice consumption is high in affected regions. In flooded rice fields, Cd uptake risk is low (Uraguchi and Fujiwara, 2012), but the trend is for more rice fields to become aerobic due to erratic rain or scarce water resources. Therefore, the risk of Cd accumulation will increase with more aerobic water management, particularly in Cd contaminated areas. To mitigate this, it is essential to develop a low Cd accumulating cultivar by down-regulating the expression of endogenous genes involved in Cd uptake and/or translocation by identifying a genetic marker and subsequently introgressing the trait into the popular varieties through marker assisted breeding. The latter approach has been validated in the field using the dysfuntional*OsNRAMP5* mutant (Ishikawa et al., 2012). It significantly decreases root Cd uptake and Cd content in the straw and grain, apparently without decreasing Fe uptake in root, shoot, and straw (Ishimaru et al., 2012; Sasaki et al., 2012). As we continue to identify new pathways to biofortification of rice with Fe and Zn, it is critical to examine the potential for each biofortification mechanism to affect Cd uptake.

## Conflict of Interest Statement

The authors declare that the research was conducted in the absence of any commercial or financial relationships that could be construed as a potential conflict of interest.
